# Folate Intake and Ovarian Cancer Risk among Women with Endometriosis: A Case–Control Study from the Ovarian Cancer Association Consortium

**DOI:** 10.1158/1055-9965.EPI-23-0121

**Published:** 2023-05-23

**Authors:** Kate Gersekowski, Torukiri I Ibiebele, for the Australian Ovarian Cancer Study Group, Jennifer A. Doherty, Holly R. Harris, Marc T. Goodman, Kathryn L. Terry, Anna H. Wu, Elisa V. Bandera, Bo Qin, Jue-Sheng Ong, Jonathan P. Tyrer, Suzanne C. Dixon-Suen, Francesmary Modugno, Harvey A Risch, Penelope M. Webb

**Affiliations:** 1 Gynaecological Cancers Group, Population Health Program, QIMR Berghofer Medical Research Institute, Brisbane, Queensland, Australia.; 2 Huntsman Cancer Institute, Department of Population Health Sciences, University of Utah, Salt Lake City, Utah.; 3 Program in Epidemiology, Division of Public Health Sciences, Fred Hutchinson Cancer Center, Seattle, Washington.; 4 Department of Epidemiology, School of Public Health, University of Washington, Seattle, Washington.; 5 Cancer Prevention and Control Program, Samuel Oschin Comprehensive Cancer Institute, Cedars-Sinai Medical Center, Los Angeles, California.; 6 Community and Population Health Research Institute, Department of Biomedical Sciences, Cedars-Sinai Medical Center, Los Angeles, California.; 7 Obstetrics and Gynecology Epidemiology Center, Brigham and Women's Hospital and Harvard Medical School, Boston, Massachusetts.; 8 Harvard T.H. Chan School of Public Health, Boston, Massachusetts.; 9 Department of Population and Public Health, Keck School of Medicine, University of Southern California, Los Angeles, California.; 10 Cancer Epidemiology and Health Outcomes, Rutgers Cancer Institute of New Jersey, New Brunswick, New Jersey.; 11 Statistical Genetics Lab, QIMR Berghofer Medical Research Institute, Brisbane, Queensland, Australia.; 12 Department of Oncology, University of Cambridge, Cambridge, UK.; 13 Institute for Physical Activity and Nutrition, School of Exercise and Nutrition Sciences, Deakin University, Geelong, Victoria, Australia.; 14 Cancer Epidemiology Division, Cancer Council Victoria, Melbourne, Victoria, Australia.; 15 Department of Epidemiology, University of Pittsburgh Graduate School of Public Health, Pittsburgh, Pennsylvania.; 16 Division of Gynecologic Oncology, Department of Obstetrics, Gynecology and Reproductive Sciences, University of Pittsburgh School of Medicine, Pittsburgh, Pennsylvania.; 17 Women's Cancer Research Center, Magee-Womens Research Institute and Hillman Cancer Center, Pittsburgh, Pennsylvania.; 18 Chronic Disease Epidemiology, Yale School of Public Health, New Haven, Connecticut.; 19 University of Queensland, School of Public Health, Brisbane, Queensland, Australia.

## Abstract

**Background:**

Although folate intake has not been associated with an increased risk of ovarian cancer overall, studies of other cancer types have suggested that high folate intake may promote carcinogenesis in precancerous lesions. Women with endometriosis (a potential precancerous lesion) have an increased risk of developing ovarian cancer; however, whether high folate intake increases risk in this group is unknown.

**Methods:**

We conducted a pooled analysis of six case–control studies from the Ovarian Cancer Association Consortium to investigate the association between folate intake and risk of ovarian cancer among women with and without self-reported endometriosis. We included 570 cases/558 controls with and 5,171/7,559 without endometriosis. We used logistic regression to estimate odds ratios (OR) and 95% confidence intervals for the association between folate intake (dietary, supplemental, and total) and ovarian cancer risk. Finally, we used Mendelian randomization (MR) to evaluate our results using genetic markers as a proxy for folate status.

**Results:**

Higher dietary folate intake was associated with an increased risk of ovarian cancer for women with endometriosis [OR, 1.37 (1.01–1.86)] but not for women without endometriosis. There was no association between supplemental folate intake and ovarian cancer risk for women with or without endometriosis. A similar pattern was seen using MR.

**Conclusions:**

High dietary folate intake may be associated with an increased risk of ovarian cancer among women with endometriosis.

**Impact:**

Women with endometriosis with high folate diets may be at increased risk of ovarian cancer. Further research is needed on the potential cancer-promoting effects of folate in this group.

## Introduction

Endometriosis is an estrogen-dependent, chronic inflammatory gynecologic condition characterized by endometrial-like tissue that grows outside the uterus and often presents as pelvic pain or infertility. It affects an estimated 10% of reproductive-age women (1); however, this is likely to be an underestimate of the true prevalence due to the difficulty in diagnosing the condition (2). Women with endometriosis have a 2- to 3-fold (3, 4) higher risk of developing endometrioid and clear cell ovarian cancers, which account for approximately 20% of all epithelial ovarian cancers (5).

Folate, a water-soluble B vitamin, plays an important role in DNA, RNA, and protein synthesis and is necessary for cell division (6). Folic acid is the synthetic form of folate used in supplements and food fortification and is better absorbed than folate from food sources due to differing bioavailability (6). The effectiveness of folate supplementation in the prevention of neural tube defects in early pregnancy led to the introduction of mandatory folate fortification of specified foods, typically flour and bread products, beginning in the United States in 1998, followed by other countries including Canada and Australia (7).

Previous epidemiologic and laboratory studies have suggested a possible dual role of folate in carcinogenesis: higher intakes may be protective for healthy epithelial cells, but may promote progression of precursor neoplastic lesions such as preneoplastic colorectal epithelial cells (8, 9). High folate intake has not been associated with an increased risk of ovarian cancer overall (10–13), although there is some evidence of effect modification by other factors including alcohol intake (14, 15). It is, however, possible that high folate intake may promote ovarian cancer in the presence of endometriosis—a known precursor lesion (16).

The aim of this study was to determine whether folate intake is associated with an increased risk of ovarian cancer, particularly the endometrioid and clear cell types, among women with and without endometriosis.

## Materials and Methods

### Participants

We pooled primary data from six case–control studies participating in the Ovarian Cancer Association Consortium (OCAC) that could provide data on folate intake and endometriosis status. This included five studies from the United States [Diseases of the Ovary and their Evaluation Study (DOV), ref. 17; Hawaii Ovarian Cancer Study (HAW), ref. 18; New England Case–Control Study of Ovarian Cancer (NEC), ref. 19; New Jersey Ovarian Cancer Study (NJO), ref. 20; Los Angeles County Case–Control Studies of Ovarian Cancer (LAC), ref. 21] and one study from Australia [Australian Ovarian Cancer Study (AUS), ref. 22].

Eligible cases included women ages 18 years or older who were diagnosed with invasive epithelial ovarian cancer (including fallopian tube and primary peritoneal cancers). Women with no prior personal history of ovarian cancer and who had at least one ovary at recruitment were included as controls. All studies obtained institutional ethics committee approval and followed recognized ethnical guidelines, including the Declaration of Helsinki, the Belmont Report, and/or the US Common Rule, and all study participants provided written informed consent.

### Inclusion and exclusion criteria

For this analysis, a total of 16,755 women (7,144 cases and 9,611 controls) from the six studies were eligible for inclusion. Women were excluded if they were missing nutrient data (*n* = 2,713) or had implausible energy intakes (*n* = 131), defined as more than three standard deviations from the mean natural logarithm of total energy among the control group for that study (23). An additional 53 women were excluded as they were missing data on endometriosis status, leaving 13,858 women (5,741 cases and 8,117 controls) included in the study population. For analyses assessing folate supplementation, women missing information (or from studies that did not collect information) on supplement use were excluded, leaving a total of 9,072 women (3,759 cases and 5,313 controls). Supplementary Fig. S1 details the exclusions applied to obtain the final study population.

### Folate intake, endometriosis status, and covariate information

Dietary information was acquired through the Multidisciplinary Ovarian Cancer Outcomes Group—a group created out of OCAC to explore, among other aims, dietary associations with survival after ovarian cancer diagnosis. Folate intake was estimated using validated food frequency questionnaires (FFQs) for AUS (24), DOV (25), HAW (26), LAC (26), NEC (27), and NJO (20). Participants were asked to report their usual frequency of consumption of a range of food items (range, 120–200 on the various FFQs) in the year or two prior to diagnosis for cases or prior to interview for controls. This information was used to estimate nutrient intakes using Australian (AUS) or US food tables. Measurement of folate intake using FFQs has been shown to be reliable with a correlation of 0.63 compared with plasma folate levels reported for the Willett FFQ (variations of which were used by AUS and NEC; ref. 28). Four studies (AUS, HAW, NEC, and NJO) and one phase (of three) of the LAC study additionally collected information on supplement use.

Folate intake was defined using three main measures: (i) dietary folate intake including folate that occurs naturally in foods as well as folic acid from fortified foods, (ii) folate intake from supplements, and (iii) total folate intake (from both diet and supplements). For dietary folate intake, we additionally differentiated between naturally occurring folate from foods such as fruits and vegetables, and synthetic folate from foods fortified with folate, including flour. As the bioavailability of natural folate is lower than that of folic acid, we calculated dietary folate equivalents (DFE) for measures including a component of folic acid intake, whereby 1 μg of folic acid was assumed to contribute 1.7 DFE (29).

All measures of dietary folate data were energy-adjusted using the residual method (30). Dietary and total folate intake were categorized using study-specific tertile cutoff points while folic acid intake from supplements was categorized based on the recommended daily intake (RDI) of 400 μg DFE for the general population as 0, <400 and 400+ μg (Supplementary Table S1). Alcohol intake (none, <10, 10+ grams/day) was also assessed using the FFQs.

Endometriosis status was self-reported via questionnaire. Four studies (DOV, HAW, NJO, and LAC) asked if a woman was ever told by a doctor/health professional that they had endometriosis and two studies (AUS and NEC) asked if a woman had ever had endometriosis prior to the reference/diagnosis date.

Dietary data were merged with information potentially relevant to ovarian cancer risk or folate intake from the OCAC core database. These variables, which had been harmonized centrally, included case–control status, age at diagnosis (or comparable reference date for controls), education (high school or less, some college, college graduate, graduate or professional degree), smoking status prediagnosis (never, former current), body mass index (BMI) (<25, 25–29, and ≥30 kg/m^2^) measured one year (AUS, NEC, NJO, LAC) or five years prior to diagnosis or interview date (DOV and HAW), first-degree family history of breast or ovarian cancer, oral contraceptive pill (OCP) use, parity, breastfeeding history, tubal ligation, endometriosis status, aspirin and nonsteroidal anti-inflammatory drug (NSAID) use. Clinical information included histotype (high-grade serous, low-grade serous, mucinous, endometrioid, clear cell, and other).

### Statistical analyses

Logistic regression models were used to estimate odds ratios (OR) and 95% confidence intervals (CI) for the association between the three main measures of folate intake and ovarian cancer risk, separately for women with and without endometriosis. We conducted analyses for all invasive cancers combined and then separately for endometrioid and clear cell (END/CCC) cancers, as they are most strongly linked to endometriosis, and high-grade serous cancers (HGSC). We also compared results of logistic regression models to equivalent generalized linear mixed models to allow random effects between OCAC sites.

Directed acyclic graphs (DAG) were generated *a priori* to identify potential confounders of the relationship between folate intake and ovarian cancer; these were retained in models if they altered the beta coefficients for folate intake by >10%. Based on the DAGs, all models were adjusted for age and total energy intake (log) and stratified by study site. Parity was included in the following models for women with endometriosis as its inclusion altered the folate estimates by >10%: dietary folate intake, total folate intake, and END/CCC subtype supplement analyses. Other potential confounders including education, BMI, smoking, OCP use, alcohol consumption, breastfeeding, race, family history, and fortification exposure (whether women completed the FFQ before or after the introduction of mandatory folate fortification) were not included in the final models as they did not alter the folate estimates appreciably.

To assess heterogeneity between studies, study-specific ORs comparing medium/high intake to low dietary folate intake were combined using random effects meta-analysis, and *I*^2^ and *P* values for heterogeneity (from chi-square tests) were calculated. To assess whether the folate–cancer association differed between women with and without endometriosis, we reran models including an interaction term between the folate variable and endometriosis. A *P* < 0.05 for the interaction term was considered statistically significant.

To assess whether any association between folate intake (medium/high vs. low) and ovarian cancer risk was modified by other factors, we stratified by potential modifiers. These included alcohol use (none, <10 g/day, 10+ g/day), BMI (<25, 25–29, ≥30 kg/m^2^), folate fortification status, NSAID and aspirin use [regular use (at least once per week vs. less often)]. These variables were chosen because they can interfere with the bioavailability of folate (as reported for alcohol; ref. 31) or affect inflammation (aspirin and NSAIDs, ref. 32, and as suggested for BMI, ref. 33). Factors related to inflammation were investigated as folate may play a role in inflammatory processes (34), and endometriosis is an inflammatory condition, so it is possible any associations may be modified by pro- or anti-inflammatory factors.

In *post hoc* analyses, we also examined the association between glycemic index (GI), glycemic load (GL), and intake of grains (total, whole and refined)—which are likely to include a high proportion of folate-fortified foods—and ovarian cancer risk. This was to assess whether associations seen for dietary folate, particularly for the synthetic component, were potentially due to the types of foods that are fortified rather than folate itself. Models were run for women with and without endometriosis and GI, GL, and grain intake were categorized using study-specific tertile cutoff points. We used chi-squared tests to assess associations between dietary folate intake and grain intake, GL and GI.

Analyses were performed using SAS version 9.4 (SAS Institute) and Stata version 15 (StataCorp LP).

### Mendelian randomization

Given possible issues with recall bias and dietary assessment in case–control studies, we also used Mendelian randomization (MR) to evaluate this association. Although we knew this would be underpowered for women with endometriosis, our primary goal was to determine if results were consistent with those from the observational analyses.

We used two-sample MR to assess the associations between folate and ovarian cancer risk using genetic markers as a proxy for serum folate levels. We used publicly available summary data for 4 single-nucleotide polymorphisms (SNP) associated with serum folate levels (predicting 1.3% variance) in the largest published genome-wide association study (GWAS) to date (ref. 35; Supplementary Table S2). Summary estimates for the association between the SNPs and ovarian cancer were not available by endometriosis status, so we estimated these using individual level data from 1,740 women with endometriosis and 19,145 women without endometriosis from 18 OCAC studies including the six studies in the dietary analysis. DNA samples had been genotyped as previously described (36). We estimated the association between each SNP and ovarian cancer risk by fitting logistic regression models adjusted for the participants’ study country of origin and ancestral principal components (between 1 and 9 depending on the genotyping platform) to account for population structure (36). All women were of genetically determined European ancestry.

We used the beta coefficients and standard errors for the SNP–folate and SNP–ovarian cancer associations to estimate ORs and 95% CIs for the effect of folate on ovarian cancer. Estimates were obtained for each SNP by dividing the SNP–outcome association by its SNP–folate association (Wald ratio).The individual SNP estimates were then combined using an inverse-variance weighted MR model (37).

We undertook sensitivity analyses to assess potential violations of the MR assumptions, including MR-Egger (38), weighted-median MR (39), and MR-PRESSO (40). We calculated Cochran's Q-statistic for between-SNP heterogeneity of effects. We checked whether SNPs were associated with other relevant traits using the NHGRI-EBI GWAS Catalog (41) and PhenoScanner (42, 43). Analyses were performed using the MendelianRandomization package (44) and MR-PRESSO package (40) implemented in the R software (R Foundation for Statistical Computing).

### Data availability

Data described in the article cannot be made publicly available due to privacy and ethical limitations imposed by the original studies in which these data were collected, but can be shared upon approval of a data request form by the OCAC Data Access Coordinating Committee and with appropriate human subjects approval and data transfer agreements.

## Results

Table 1 shows the characteristics of cases and controls with and without endometriosis in the observational analysis. The majority of cases and controls were white and, as expected, compared with controls, cases were more likely have a shorter duration of OCP use, more likely to be nulliparous, less likely to have breastfed and less likely to have had tubal ligation. Women who reported a previous diagnosis of endometriosis were younger, more likely to be nulliparous and had a longer duration of OCP use than those without endometriosis. Cases with endometriosis were more likely to have endometrioid or clear cell cancers (38.1%) than those without endometriosis (20.6%).

**Table 1.** Characteristics of cases and controls with and without endometriosis.

	Endometriosis		Without endometriosis	
	Cases (*N* = 570)	Controls (*N* = 558)	Cases (*N* = 5,171)	Controls (*N* = 7,559)
Age, mean (SD)	54.0 (9.7)	53.5 (10.7)	57.1 (11.3)	55.6 (12.5)
Ethnicity				
White	476 (83.8)	481 (86.5)	4,428 (85.6)	6,480 (86.0)
Asian	53 (9.3)	40 (7.2)	387 (7.5)	467 (6.2)
Other	39 (6.9)	35 (6.3)	357 (6.9)	590 (7.8)
Education, *n* (%)				
High school or less	130 (22.9)	135 (24.2)	1,949 (38.0)	2,325 (30.8)
Some university	197 (34.7)	187 (33.5)	1,570 (30.6)	2,410 (31.9)
University graduate	104 (18.3)	128 (22.9)	894 (17.4)	1,507 (20.0)
Graduate or prof degree	137 (24.1)	108 (19.4)	711 (13.9)	1,305 (17.3)
Smoking, *n* (%)				
Never	333 (58.5)	274 (49.1)	2,817 (54.9)	4,135 (54.8)
Former	174 (30.6)	210 (37.6)	1,656 (32.3)	2,449 (32.4)
Current	62 (10.9)	74 (13.3)	657 (12.8)	966 (12.8)
BMI (kg/m^2^), mean (SD)	26.1 (5.9)	25.6 (5.6)	26.4 (6.1)	26.0 (5.6)
Alcohol (g/day), *n* (%)				
None	125 (21.9)	96 (17.2)	1,331 (25.7)	1,565 (20.7)
<10	335 (58.8)	344 (61.6)	2,864 (55.4)	4,338 (57.4)
10+	110 (19.3)	118 (21.1)	976 (18.9)	1,656 (21.9)
First-degree relative with Br/OvCa				
No	420 (83.0)	402 (82.9)	3,342 (76.4)	5,319 (82.5)
Yes	86 (17.0)	83 (17.1)	1,035 (23.6)	1,129 (17.5)
OCP use, *n* (%)				
Never	168 (29.5)	123 (22.2)	2,294 (44.5)	2,391 (31.7)
<5 years	232 (40.8)	229 (41.3)	1,685 (32.7)	2,533 (33.6)
5–9.9 years	100 (17.6)	100 (18.0)	647 (12.6)	1,310 (17.4)
10+ years	69 (12.1)	103 (18.6)	527 (10.2)	1,315 (17.4)
Parity, *n* (%)				
0	221 (38.8)	130 (23.3)	1,241 (24.0)	1,187 (15.7)
1	104 (18.3)	90 (16.1)	697 (13.5)	985 (13.0)
2	141 (24.8)	176 (31.5)	1,465 (28.4)	2,380 (31.5)
3	64 (11.2)	104 (18.6)	988 (19.1)	1,649 (21.8)
4+	39 (6.9)	58 (10.4)	776 (15.0)	1,358 (18.0)
Breastfed (parous women only), *n* (%)				
No	190 (45.7)	158 (33.5)	1,770 (42.0)	2,249 (33.5)
Yes	224 (54.9)	314 (66.5)	2,435 (57.8)	4,451 (66.4)
Don't know	2 (0.5)	0 (0.0)	11 (0.3)	4 (0.1)
Tubal ligation, *n* (%)				
No	379 (85.6)	348 (79.1)	3,524 (84.2)	4,984 (78.3)
Yes	64 (14.4)	92 (20.9)	660 (15.8)	1,380 (21.7)
Aspirin use, *n* (%)				
Nonregular use (<once/week)	303 (80.2)	316 (82.7)	2,883 (83.4)	4,415 (82.4)
Regular use (≥once/week)	75 (19.8)	66 (17.3)	574 (16.6)	941 (17.6)
Nonaspirin NSAID use, *n* (%)				
Nonregular use (<once/week)	275 (73.1)	274 (71.7)	2,762 (79.9)	4,228 (78.9)
Regular use (≥once/week)	101 (26.9)	108 (28.3)	693 (20.1)	1,130 (21.1)
Total dietary folate^a^, *n* (%)				
Lowest tertile	158 (27.7)	200 (35.8)	1,731 (33.5)	2,528 (33.4)
Medium	205 (36.0)	182 (32.6)	1,738 (33.6)	2,498 (33.0)
Highest tertile	207 (36.3)	176 (31.5)	1,702 (32.9)	2,533 (33.5)
Folic acid from supplementation^b^, *n* (%)				
0mcg	176 (47.3)	169 (47.9)	1,769 (52.2)	2,589 (52.2)
0–400 μg	51 (13.7)	50 (14.2)	471 (13.9)	682 (13.8)
400+ μg	145 (39.0)	134 (38.0)	1,147 (33.9)	1,689 (34.1)
Not collected/missing	198	205	1,784	2,599

Note: Numbers may not sum to the total because of missing data.

Abbreviations: BMI, body mass index; Br/OvCa, breast/ovarian cancer; NSAID, nonsteroidal anti-inflammatory drug; OCP, oral contraceptive pill.

^a^Site-specific tertiles of total dietary folate intake (DFE).

^b^Dietary folate equivalents.

Estimated dietary folate intake and supplement use varied across study sites (Table 2). Folate intake was higher after the introduction of fortification programs and supplement use was more common in the USA than in Australia.

**Table 2.** Median folate intake and mandatory folate fortification status, OCAC studies.

			Dietary folate intake		Folate intake from supplements		
Study	Recruitment year	Fortification status (year started)	*N*	Median intake^a^	*N* ^b^	% users	Median intake^c^
**AUS**	**2002–2005**	**Pre (2009)**	**2,602**	**423**	**2,595**	**29**	**180**
**DOV**	**2002–2009**	**Post (1998)**	**2,755**	**473**	**0**	**N/A**	**N/A**
**HAW**	**1993–2008**	**Mixed (1998)**	**1,796**	**380**	**1,433**	**54**	**400**
	1993**–**1998	Pre	1,040	321	775	51	400
	1999**–**2008	Post	756	493	658	57	400
**NEC**	**1992–2003**	**Mixed (1998)**	**3,451**	**432**	**3,451**	**54**	**400**
	1992**–**1998	Pre	877	305	877	34	400
	1999**–**2003	Post	2,574	469	2,574	61	400
**NJO**	**2002–2008**	**Post (1998)**	**586**	**390**	**586**	**67**	**400**
**LAC**	**1994–2004**	**Mixed (1998)**	**2,668**	**427**	**1,007**	**59**	**636**
Phase I	1994**–**1999	Pre	1,269	345	N/A	N/A	N/A
Phase II	1994**–**1998	Pre	392	354	N/A	N/A	N/A
Phase III	2000**–**2004	Post	1,007	627	1,007	59	626

Abbreviations: N/A, not applicable; OCAC, Ovarian Cancer Association Consortium.

^a^Dietary folate equivalents in micrograms.

^b^Number of participants with data available on supplement use.

^c^Median intake among supplement users only.

Table 3 shows the associations between folate intake and risk of invasive epithelial ovarian cancer for women with and without endometriosis. Among women with endometriosis, there was a suggestion that higher dietary folate intake was associated with an increased risk of invasive ovarian cancer [tertile 2 (T2): OR 1.29 (95% CI, 0.95–1.75); T3: 1.37 (1.01–1.86) vs. T1; *P*_trend_ 0.045]. No increased risk was seen for women without endometriosis. Among women with endometriosis, the association was stronger for synthetic folate from dietary sources [OR for T2: 1.73 (1.17–2.56); T3: 1.36 (0.92–1.99)]; there was also a suggestion that synthetic folate was associated with an increased risk of ovarian cancer for women without endometriosis [OR for T2: 1.17 (1.04–1.30); T3: 1.10 (0.98–1.23)]. Conversely, an inverse association was seen for naturally occurring folate (*P*_trend_ < 0.001) for women without endometriosis. There were no significant associations for folic acid from supplements or total folate intake (diet and supplements) for women in either group. The patterns did not differ when we considered endometrioid/clear cell cancer and HGSC separately (Supplementary Table S3). There was little difference in results when we used generalized linear mixed models.

**Table 3.** Association between folate intake and risk of invasive epithelial ovarian cancer, by endometriosis status.

	Endometriosis		Without endometriosis	
Folate variable	Cases/controls	OR (95% CI)^a^	Cases/controls	OR (95% CI)^a^
**Dietary folate intake (tertile)** **^b^**				
** **Low	158/200	1.00 (ref)	1,731/2,528	1.00 (ref)
** **Medium	204/182	1.29 (0.95–1.75)	1,738/2,498	0.99 (0.91–1.09)
** **High	207/176	1.37 (1.01–1.86)	1,702/2,533	0.96 (0.88–1.04)
**Natural folate from diet (tertile)** ^b^ ^,^ ^c^				
** **Low	112/127	1.00 (ref)	1,077/1,522	1.00 (ref)
** **Medium	117/116	1.08 (0.74–1.58)	1,058/1,550	0.94 (0.84–1.05)
** **High	126/116	1.10 (0.76–1.61)	976/1619	0.82 (0.73–0.92)
**Synthetic folate (folic acid) from fortified diet items (tertile)** ^b^ ^,^ ^c^				
** **Low	92/125	1.00 (ref)	1,005/1,616	1.00 (ref)
** **Medium	131/106	1.73 (1.17–2.56)	1,084/1,519	1.17 (1.04–1.30)
** **High	132/128	1.36 (0.92–1.99)	1,022/1,556	1.10 (0.98–1.23)
**Folic acid from supplements** ^b^ ^,^ ^d^				
None (0 μg DFE)	176/168	1.00 (ref)	1,769/2,589	1.00 (ref)
** **Low (<400 μg DFE)	80/74	1.07 (0.73–1.58)	679/961	1.05 (0.93–1.18)
** **High (400+ μg DFE)	116/110	1.06 (0.74–1.51)	939/1,410	0.99 (0.89–1.11)
**Total folate intake (from diet and supplementation; tertile)** **^b^**				
** **Low	115/113	1.00 (ref)	1,111/1,683	1.00 (ref)
** **Medium	117/105	0.97 (0.66–1.44)	1,132/1,672	1.00 (0.90–1.12)
** **High	139/135	0.96 (0.66–1.40)	1,144/1,605	1.02 (0.92–1.14)

Abbreviations: CI, confidence interval; DFE, dietary folate equivalents; μg, micrograms; OR, odds ratio.

^a^All models were adjusted for age (categorical, 10-year age groups), log(energy intake), and stratified by site. Models for dietary folate intake and total intake for women with endometriosis were additionally adjusted for parity. Adjusting for parity in other models made no appreciable difference to estimates.

^b^Study-specific tertiles (low, medium, and high) were used for all models except for folic acid from supplementation, which used cutoff points based on the folate RDI (0 μg, <400 μg, and 400+ μg). DFEs were used for measures that included a component of folic acid intake.

^c^Includes only participants from AUS, DOV, NEC, and NJO due to data availability. Natural folate from diet included folate intake from food sources where folate naturally occurs, such as fruits and vegetables. Synthetic folate from diet includes intake from food sources which were fortified with synthetic folate, such as flour and bread products.

^d^Folic acid from supplements includes any folate intake from supplements, including multivitamins. Participants from DOV and the first two phases of LAC were not included due to data availability.

**Figure 1.**
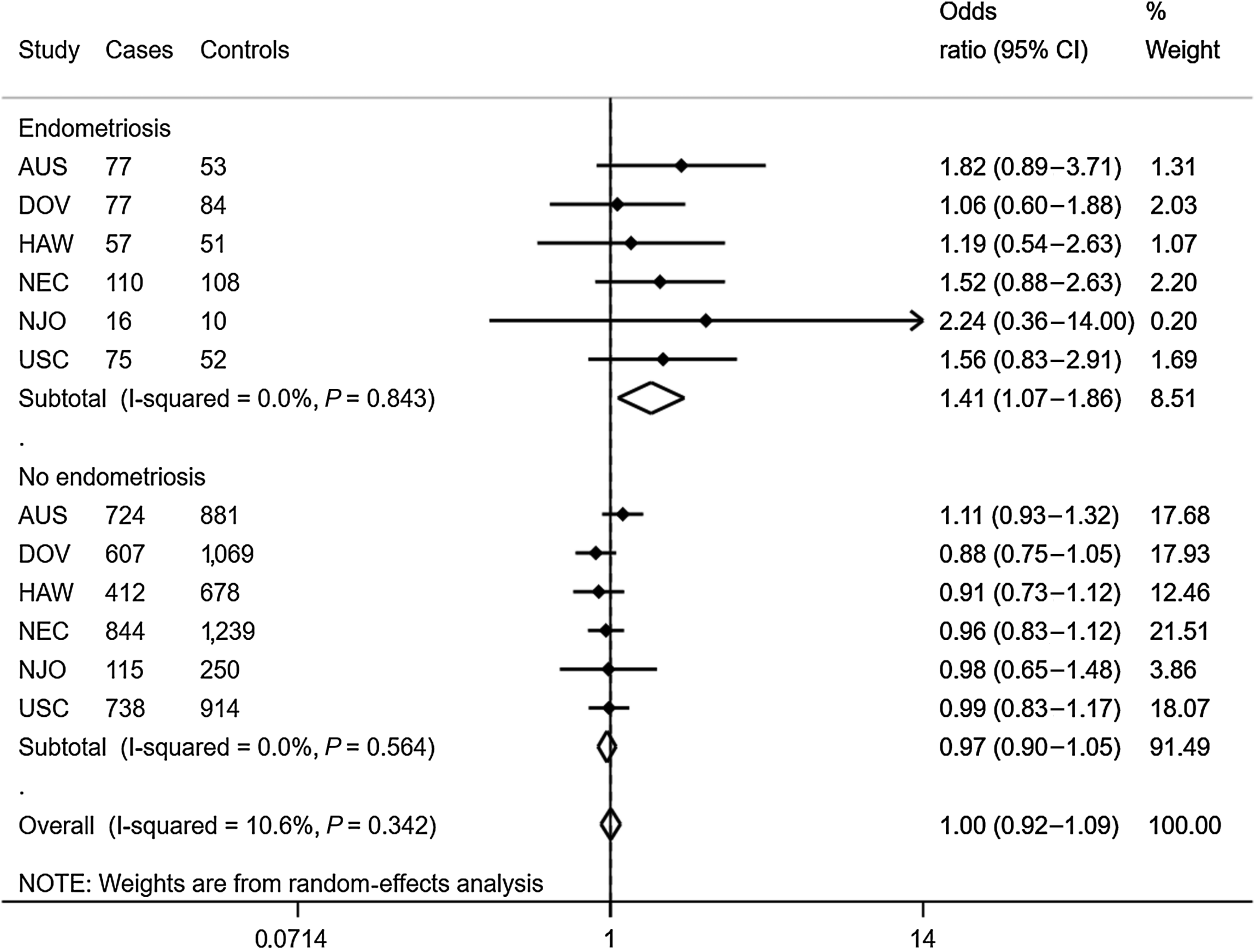
Association between dietary folate intake and risk of ovarian cancer, by study site and endometriosis status. Forest plots depicting site-specific associations between medium/high folate intake and ovarian cancer separately for women with and without endometriosis. Logistic regression was used to estimate the odds ratios and 95% CIs; all models were adjusted for age and total energy intake (log). Note: medium and high tertiles were combined and compared with low intake.

The associations between dietary folate intake and ovarian cancer risk were consistent across the study sites, with a 41% increased risk of ovarian cancer (95% CI, 1.07–1.85, *I*^2^ = 11%, *P* = 0.3) associated with medium/high dietary folate intake among women with endometriosis, but no association (OR, 0.97; 0.90–1.05) among those without endometriosis (*P*_interaction_ = 0.0001; Fig. 1).

**Figure 2.**
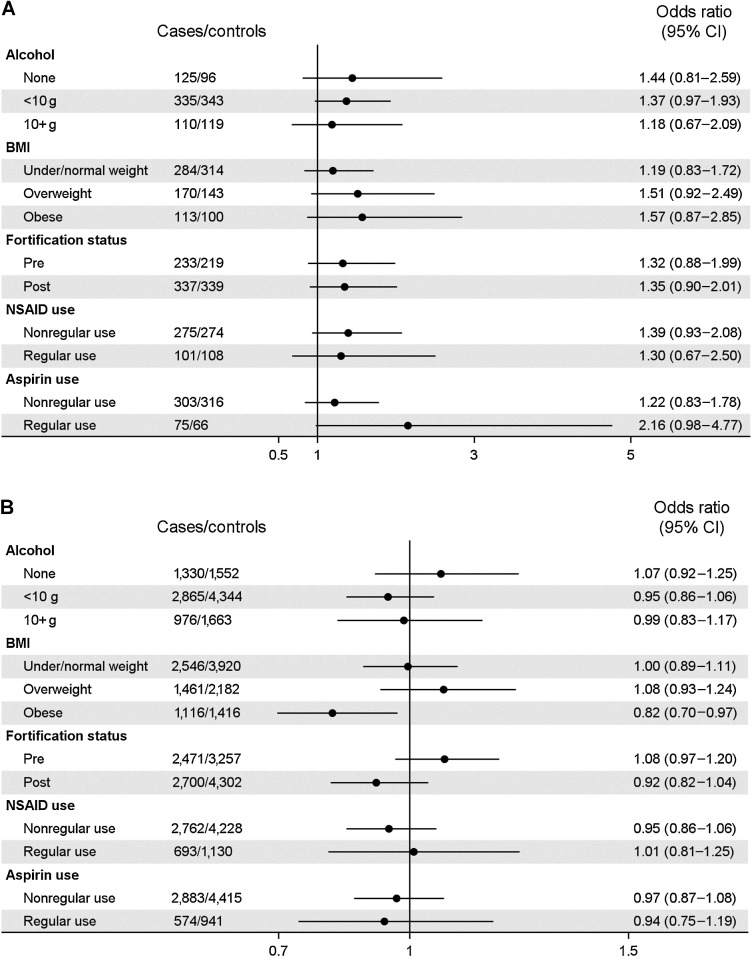
Association between (**A**) dietary folate intake and (**B**) folate/folic acid intake from supplements, and risk of ovarian cancer among women with endometriosis, stratified by potential effect modifiers. Forest plots depicting the association between (**A**) dietary folate intake and (**B**) supplemental folate intake and ovarian cancer for women with endometriosis, stratified by potential effect modifiers including alcohol intake, BMI, folate fortification status, NSAID use and aspirin use. Abbreviations: BMI, body mass index; CI, confidence interval; NSAID, nonsteroidal anti-inflammatory drug; OR, odds ratio. (i) For dietary folate intake, medium/high vs. low tertiles. For supplement folate intake, any (>0 μg) vs. none (0 μg). (ii) All models were adjusted for age (categorical, 10 years) and log(energy intake) and stratified by site. Models for dietary folate intake were additionally adjusted for parity. Adjusting for parity in other models made no appreciable difference to estimates.

Figure 2 shows the associations between folate intake (dietary and supplemental) and risk of ovarian cancer stratified by alcohol intake, BMI, fortification status, NSAID, and aspirin use, for women with endometriosis. Among women who took NSAIDs at least once per week, supplemental folate intake was associated with an increased risk of ovarian cancer (OR 2.29; 95% CI, 1.08–4.84), whereas among nonusers there was a decreased risk (OR, 0.65; 95% CI, 0.42–0.98). This difference was not seen for dietary folate intake or total folate intake. A similar increased risk was seen for dietary folate among regular aspirin users (OR, 2.16; 95% CI, 0.98–4.77). There was no strong evidence of effect modification for the other variables. For women without endometriosis, the only evidence of effect modification was for folate intake from supplements and alcohol intake (Supplementary Fig. S2).

*Post hoc* analyses (Supplementary Table S4) to assess whether observed associations were potentially due to the types of foods fortified, rather than folate itself, showed no association between grain intake (total, refined, or whole) and ovarian cancer in women with or without endometriosis. Among those without endometriosis, higher GI and GL but not grain intake were associated with an increased risk of ovarian cancer (GI, high intake: OR 1.31 (1.13–1.53); GL, high intake: OR 1.38 (1.20–1.58)], which is consistent with published literature (45). This was not observed among women with endometriosis. Dietary folate intake was significantly associated with grain intake (total, refined, and whole), GL and GI (all *P* < 0.005).

### Mendelian randomization

Although the number of women with endometriosis was relatively small, so the SNP–cancer estimates for this group were imprecise, we saw the same pattern with a suggested increased risk of ovarian cancer for higher genetically predicted folate levels in women with endometriosis [OR, 2.22 (0.80–6.17) per 1 standard deviation higher folate] but no association among those without endometriosis [OR, 0.90 (0.60–1.34); Supplementary Fig. S3]. Results from the sensitivity analyses were broadly consistent (Supplementary Table S5) and no outlying SNPs were identified using MR-PRESSO.

## Discussion

Our results, from both self-reported intake and genetically predicted measures of serum folate, support our *a priori* hypothesis that higher folate intake increases the risk of ovarian cancer among women with endometriosis but not those without. There was also a suggestion that higher intake of synthetic folate, folic acid added to foods during fortification, but not naturally occurring folate in foods was associated with increased risk. However, this may have been a chance finding as we did not see any association with folic acid intake from supplements.

Previous reports (including one each from AUS, ref. 10 and NEC, ref. 11) and a meta-analysis with 12 studies (including AUS and NEC) have shown no association between high folate intake and risk of ovarian cancer (12, 13), but due to the relatively low prevalence of endometriosis in the population (approximately 10%; ref. 1), most women included in these studies would not have had endometriosis. Our results for women without endometriosis are consistent with this. Two previous studies (14, 15) have suggested possible effect modification with alcohol intake, with the association between folate and reduced ovarian cancer risk limited to those with higher alcohol intake. Our results suggested that there was variation by alcohol intake for both women with and without endometriosis, but this was limited to supplement use and suggested increased risk at higher alcohol intakes. However, it is important to note that alcohol intake was low across the study populations so we had limited power to assess effect modification with high intakes. Our observations that the associations might also vary by aspirin or NSAID use in women with endometriosis are interesting but, given the lack of consistency between associations for dietary and supplemental intake and for aspirin and other NSAIDs, it is hard to draw any definitive conclusions.

Folate may have dual effects on cancer development and progression. Epidemiologic and laboratory studies in colorectal cancer have shown that, in normal cells, folate deficiency may lead to cancer progression through impaired DNA repair and increased mutations, while higher levels may be protective (9). However, *in vitro* and *in vivo* studies have shown that in the presence of established premalignant colorectal lesions with rapidly replicating cells, folate supplementation may accelerate progression to cancer by promoting further proliferation and progression (9). Although observations found in colorectal cancer may not necessarily be relevant for ovarian cancer, our results support a similar hypothesis.

Strengths of this study include the large sample size and pooled design. The retrospective self-reporting of dietary intake is a limitation and may potentially introduce measurement error. However, it is unlikely this would differ by endometriosis status, so it is unlikely to explain the observed difference in the ORs between women with and without endometriosis. It remains possible that the observed associations are due to unknown or unmeasured confounding factors; however, our model accounts for known risk factors associated with folate intake and risk of ovarian cancer. Associations with dietary folate intake could also be due to the kinds of foods that are fortified (e.g., bread). However, we also saw no association between grain intake, GI and GL, and ovarian cancer risk for women with endometriosis, so it is unlikely that the observed associations are due to food type rather than folate intake. We saw a similar pattern, although the individual estimates were not statistically significant, using MR. This consistency adds confidence that overall findings are unlikely to be due to bias or confounding.

It is possible that the null associations for supplement use could be due to residual confounding by socioeconomic factors that might affect access to and use of supplements (46) or potential misclassification by participants in the type or brand of supplement used. Additionally, we were unable to directly assess the impact of duration of supplement use, and it is possible that any increased risk may be observed only in long-term users.

A further limitation is that there are likely to be women with undiagnosed endometriosis in the no endometriosis group. Additionally, endometriosis status was self-reported and laparoscopic confirmation was not required, so some women may have been misdiagnosed. However, a recent study showed good agreement (84%) between self-reported endometriosis and medical records in general (47). Both situations would tend to make the groups look more similar leading to underestimates of the difference between women with and without endometriosis.

Age at endometriosis diagnosis was not routinely collected across studies, so we were unable to assess the relevance of timing of endometriosis diagnosis in relation to folate intake. However, in studies that had information, most women were diagnosed with endometriosis prior to dietary assessment (median = 19 years). Using MR, although underpowered, helped to address this issue by providing an unbiased estimate of folate intake unrestricted to a particular time point.

It is important to note that ovarian cancer is more common in older women; thus, the majority of women in this analysis were postmenopausal. Given the established benefits of folate supplementation during pregnancy for preventing neural tube defects and the rarity of ovarian cancer in women of reproductive age, women with endometriosis who plan to conceive should follow established guidelines for folic acid supplementation.

In summary, our results suggest that higher folate intake, particularly from dietary sources, may be associated with increased risk of ovarian cancer among women with endometriosis. There is a need for additional research to better understand the role of dietary and supplementary folate sources in ovarian cancer risk, especially the potential cancer-promoting effect of high folate intake in women with endometriosis.

## Supplementary Material

Supplementary Figure 1 Supplementary Figure 1 shows a flowchart of exclusions to obtain the study population, with the number of cases and controls eligible for the analysis.

Supplementary Figure 2 Supplementary Figure 2 shows two forest plots depicting the association between (A) dietary folate intake and (B) supplemental folate intake and ovarian cancer for women with endometriosis, stratified by potential effect modifiers

Supplementary Figure 3 Supplementary Figure 3 shows scatter plots with the genetic association with folate on the x-axis and the genetic association with ovarian cancer on the y-axis, for (A) women with and (B) without endometriosis. The regression line for the inverse variance weighted Mendelian randomization method is shown.

Supplementary Table 1 Supplementary Table 1 shows the range of folate intake included in each tertile, by OCAC study site.

Supplementary Table 2 Supplementary Table 2 shows the single nucleotide polymorphisms (SNPs) used as instruments for folate level in the Mendelian randomization analysis.

Supplementary Table 3 Supplementary Table 3 shows the associations between folate intake (dietary, supplemental, total) and ovarian cancer by endometriosis status, and by histological subtype (endometrioid/clear cell cancers and high grade serous cancers).

Supplementary Table 4 Supplementary Table 4 shows the association between glycemic index, glycemic load, grain intake and risk of ovarian cancer for women with and without endometriosis.

Supplementary Table 5 Supplementary Table 5 shows the results from the different Mendelian randomization methods investigating the association between genetically predicted folate and risk of ovarian cancer, by endometriosis status
