# Supplementary material for: Folate Intake and Ovarian Cancer Risk among Women with Endometriosis: A Case–Control Study from the Ovarian Cancer Association Consortium
Source: Cancer Epidemiol Biomarkers Prev. 2023 May 23;32(8):1087–96. doi: 10.1158/1055-9965.EPI-23-0121 (PMC10390886; doi:10.1158/1055-9965.EPI-23-0121)
Supplement: Supplementary Figure 1 — shows a flowchart of exclusions to obtain the study population, with the number of cases and controls eligible for the analysis. [file epi-23-0121_supplementary_figure_1_suppsf1.pdf]

Supplementary Figure 1: Flowchart of exclusions to obtain study population
Supplementary Figure 1 shows the number of cases and controls eligible for the analysis, and the number excluded for each analysis.

Supplementary Figure 1

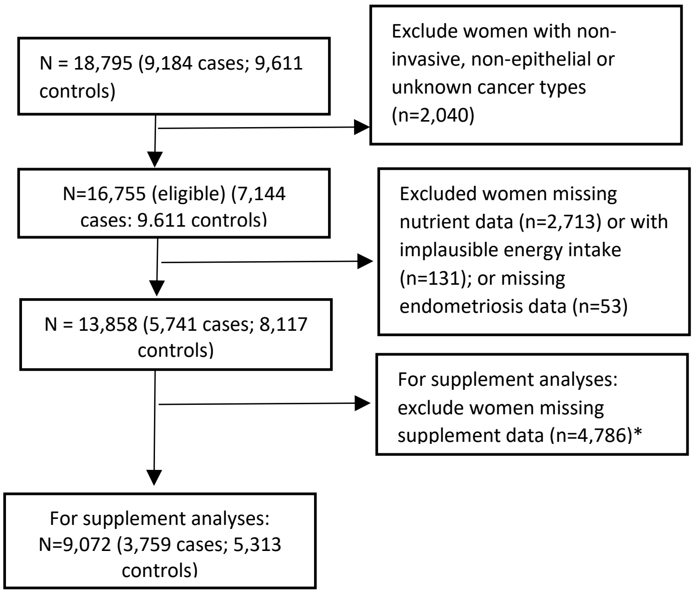

* Supplement data were not collected in DOV (n=2,755) or the first two phases of LAC (n=1,661). Of the remaining women missing supplement data, 363 were from HAW and 7 from AUS.
