# Supplementary material for: Folate Intake and Ovarian Cancer Risk among Women with Endometriosis: A Case–Control Study from the Ovarian Cancer Association Consortium
Source: Cancer Epidemiol Biomarkers Prev. 2023 May 23;32(8):1087–96. doi: 10.1158/1055-9965.EPI-23-0121 (PMC10390886; doi:10.1158/1055-9965.EPI-23-0121)
Supplement: Supplementary Figure 2 — shows two forest plots depicting the association between (A) dietary folate intake and (B) supplemental folate intake and ovarian cancer for women with endometriosis, stratified by potential effect modifiers [file epi-23-0121_supplementary_figure_2_suppsf2.pdf]

Supplementary Figure 2: Association between (A) dietary folate intake and (B) folate intake from supplements and risk of ovarian cancer among women without endometriosis, stratified by potential effect modifiers Supplementary Figure 2 shows two forest plots depicting the association between (A) dietary folate intake and (B) supplemental folate intake and ovarian cancer for women without endometriosis, stratified by potential effect modifiers including alcohol intake, BMI, folate fortification status, NSAID use and aspirin use

Supplementary Figure 2

(A)

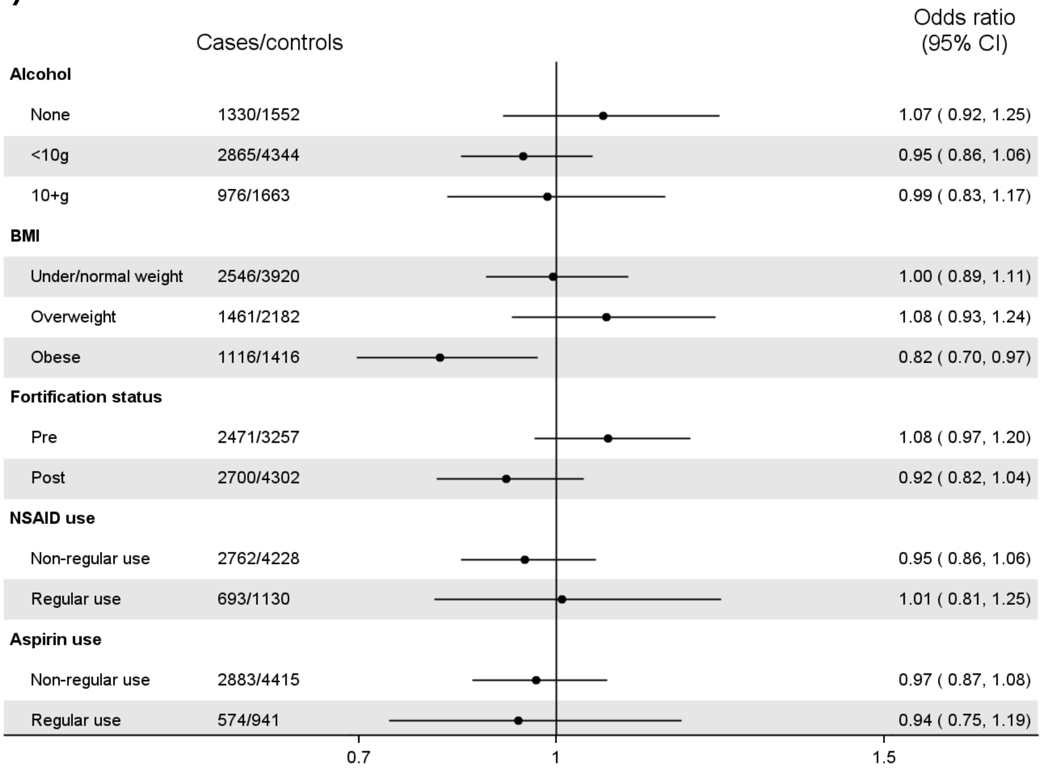

(B)

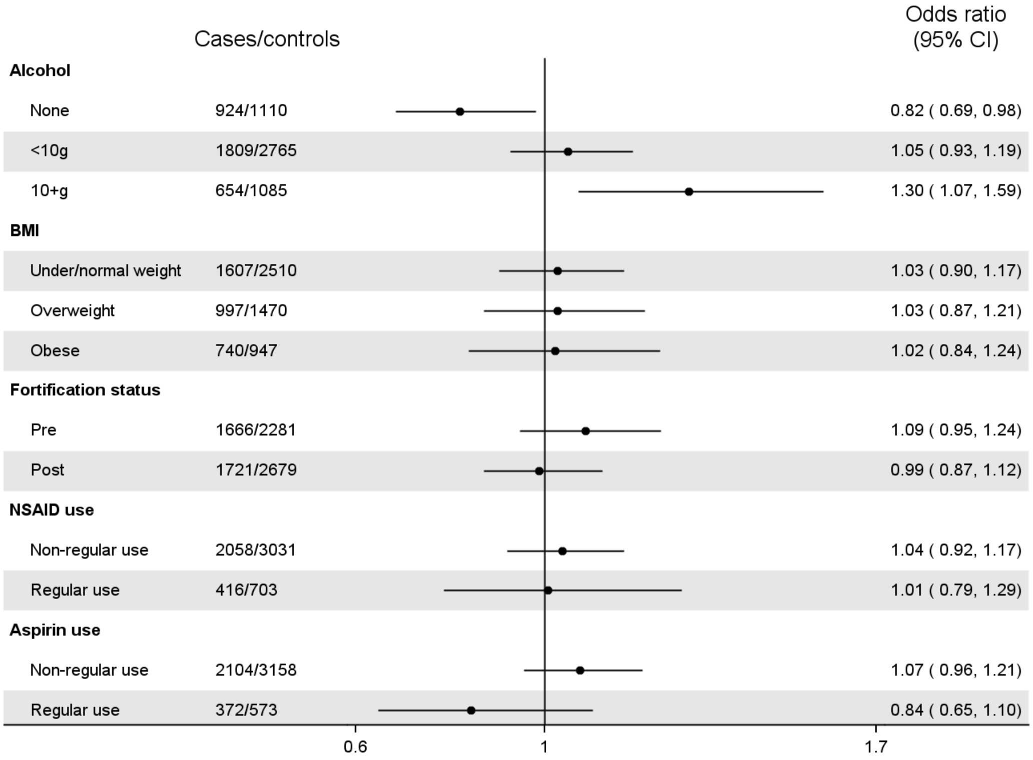

Abbreviations: BMI, body mass index; CI, confidence interval; NSAID, non-steroidal anti-inflammatory drug; OR, odds ratio.

1. For dietary folate intake, medium/high vs low tertiles. For supplement folate intake, any (>0mcg) vs none (0mcg).
2. All models were adjusted for age (categorical, 10 years), log(energy intake) and stratified by site. Models for dietary folate intake were additionally adjusted for parity. Adjusting for parity in other models made no appreciable difference to estimates
