# Supplementary material for: Folate Intake and Ovarian Cancer Risk among Women with Endometriosis: A Case–Control Study from the Ovarian Cancer Association Consortium
Source: Cancer Epidemiol Biomarkers Prev. 2023 May 23;32(8):1087–96. doi: 10.1158/1055-9965.EPI-23-0121 (PMC10390886; doi:10.1158/1055-9965.EPI-23-0121)
Supplement: Supplementary Figure 3 — shows scatter plots with the genetic association with folate on the x-axis and the genetic association with ovarian cancer on the y-axis, for (A) women with and (B) without endometriosis. The regression line for the inverse variance weighted Mendelian randomization method is shown. [file epi-23-0121_supplementary_figure_3_suppsf3.pdf]

Supplementary Figure 3: Scatter plots of SNP associations with folate (exposure) and ovarian cancer (outcome), for (A) women with and (B) without endometriosis

Supplementary Figure 3 shows scatter plots with the genetic association with folate on the x-axis and the genetic association with ovarian cancer on the y-axis, for (A) women with and (B) without endometriosis. The regression line for the inverse variance weighted Mendelian randomization method is shown.

Supplementary Figure 3

(A) Women with endometriosis **(B) Women without endometriosis**

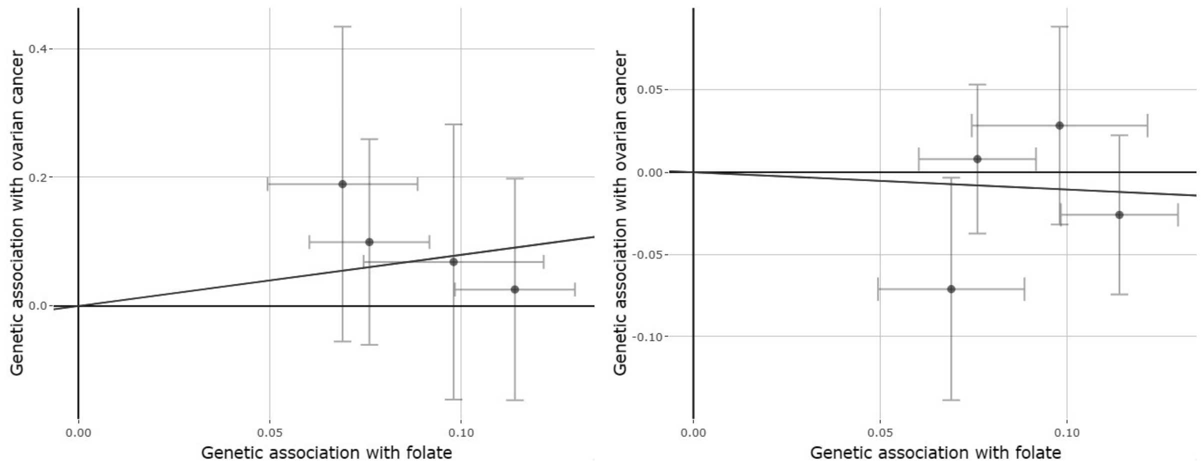

Abbreviations: SNP, single nucleotide polymorphism. Note: The regression line for the inverse variance weighted Mendelian randomization method is shown.
