# Supplementary material for: Folate Intake and Ovarian Cancer Risk among Women with Endometriosis: A Case–Control Study from the Ovarian Cancer Association Consortium
Source: Cancer Epidemiol Biomarkers Prev. 2023 May 23;32(8):1087–96. doi: 10.1158/1055-9965.EPI-23-0121 (PMC10390886; doi:10.1158/1055-9965.EPI-23-0121)
Supplement: Supplementary Table 1 — shows the range of folate intake included in each tertile, by OCAC study site. [file epi-23-0121_supplementary_table_1_suppst1.pdf]

Supplementary Table 1: Range of folate intake in tertiles, by site

	AUS	DOV	HAW	NEC	NJO	LAC
**Dietary folate intake[a]**						
Low	97–<367	140–<422	81–<321	97–<372	177–<346	90–<352
Medium	367–<490	422–<539	321–<457	372-<494	346–<445	352–<531
High	490–2376	539–2375	457–3276	494-2008	446–1362	531–2587
**Natural folate from diet [a]**						
Low	78–<276	62–<206	n.a.	59–<241	62–<184	n.a.
Medium	276–<346	206–<266	n.a.	241–<313	185–<235	n.a.
High	346–869	266–957	n.a.	313–1393	235–466	n.a.
**Synthetic folate (folic acid) from fortified diet items[a]**						
Low	0–<59	0–<180	n.a.	0–<142	3–<143	n.a.
Medium	59–<155	180–<279	n.a.	142–<225	143–<206	n.a.
High	155–2313	279–2255	n.a.	225–1683	206–1120	n.a.
**Total folate intake (from diet and supplementation)[a]**						
Low	97–<410	n.a.	100–<454	97–<471	177–<568	133–<803
Medium	410–<596	n.a.	454–<983	471–<1030	568–<1091	803–<1659
High	596–9101	n.a.	983–9933	1030–3043	1091–2531	1659–11990

[a]Dietary folate equivalents in micrograms. Abbreviations: n.a. not available; <, less than.
