# Supplementary material for: Folate Intake and Ovarian Cancer Risk among Women with Endometriosis: A Case–Control Study from the Ovarian Cancer Association Consortium
Source: Cancer Epidemiol Biomarkers Prev. 2023 May 23;32(8):1087–96. doi: 10.1158/1055-9965.EPI-23-0121 (PMC10390886; doi:10.1158/1055-9965.EPI-23-0121)
Supplement: Supplementary Table 2 — shows the single nucleotide polymorphisms (SNPs) used as instruments for folate level in the Mendelian randomization analysis. [file epi-23-0121_supplementary_table_2_suppst2.pdf]

Supplementary Table 2: Single nucleotide polymorphisms used as instruments for folate level

SNP	Candidate gene at the locus	Effect/Other allele	Beta for association with trait	SE for association with trait	Effect allele frequency
rs17421511	*MTHFR*	G/A	0.098	0.012	0.827
rs1801133	*MTHFR*	G/A	0.114	0.008	0.668
rs1999594	*MTHFR*	A/G	0.076	0.008	0.445
rs652197	*FOLR3*	C/T	0.069	0.01	0.179

Abbreviations: *FOLR3*, Folate receptor 3; *MTHFR*, methylenetetrahydrofolate reductase; SE, standard error; SNP, single nucleotide polymorphism
