# Supplementary material for: Folate Intake and Ovarian Cancer Risk among Women with Endometriosis: A Case–Control Study from the Ovarian Cancer Association Consortium
Source: Cancer Epidemiol Biomarkers Prev. 2023 May 23;32(8):1087–96. doi: 10.1158/1055-9965.EPI-23-0121 (PMC10390886; doi:10.1158/1055-9965.EPI-23-0121)
Supplement: Supplementary Table 3 — shows the associations between folate intake (dietary, supplemental, total) and ovarian cancer by endometriosis status, and by histological subtype (endometrioid/clear cell cancers and high grade serous cancers). [file epi-23-0121_supplementary_table_3_suppst3.pdf]

Supplementary Table 3: Association between folate intake and risk of ovarian cancer, by histotype and endometriosis status

Histotype	Variable	Endometriosis		Without endometriosis	
		Cases/controls	OR (95% CI)[a]	Cases/controls	OR (95% CI)[a]
**END/CCC**	**Dietary folate intake[b]**				
	Low	56/200	1.00 (ref)	368/2528	1.00 (ref)
	Medium	90/182	1.54 (1.01-2.35)	371/2498	1.04 (0.89-1.21)
	High	70/176	1.40 (0.90-2.16)	328/2533	0.90 (0.77-1.05)
**HGSC**	**Dietary folate intake[b]**				
	Low	65/200	1.00 (ref)	988/2528	1.00 (ref)
	Medium	90/182	1.37 (0.93-2.04)	996/2498	0.98 (0.88-1.08)
	High	96/176	1.57 (1.06-2.34)	1027/2533	0.99 (0.89-1.1)
**END/CCC**	**Folic acid from supplementation[b]**				
	Low (0mcg)	61/169	1.00 (ref)	341/2589	1.00 (ref)
	Medium (<400mcg)	41/74	1.55 (0.92-2.62)	145/961	1.12 (0.91-1.38)
	High (400+ mcg)	37/110	0.82 (0.49-1.39)	206/1410	1.09 (0.90-1.33)
**HGSC**	**Folic acid from supplementation[b]**				
	Low (0mcg)	77/169	1.00 (ref)	1064/2589	1.00 (ref)
	Medium (<400mcg)	31/74	0.88 (0.52-1.48)	385/961	1.00 (0.87-1.15)
	High (400+ mcg)	55/110	1.19 (0.74-1.9)	520/1410	0.90 (0.78-1.03)
**END/CCC**	**Total folate intake (diet and supplements)[b]**				
	Low	43/113	1.00 (ref)	233/1683	1.00 (ref)
	Medium	48/105	1.16 (0.68-1.99)	224/1672	0.98 (0.80-1.19)
	High	48/135	0.83 (0.49-1.40)	235/1605	1.08 (0.89-1.32)
**HGSC**	**Total folate intake (diet and supplements)[b]**				
	Low	46/113	1.00 (ref)	650/1683	1.00 (ref)
	Medium	47/105	1.01 (0.6-1.69)	643/1672	0.96 (0.84-1.09)
	High	70/135	1.19 (0.73-1.92)	676/1605	0.98 (0.86-1.12)

Abbreviations: CI, confidence interval; END/CCC, endometrial/clear cell cancers; HGSC, high grade serous cancers; mcg, micgrograms; OR, odds ratio.

[a]All models were adjusted for age (10 year age groups), log(energy intake) and stratified by site. Models for END/CCC subtype supplement intake for women with endometriosis were additionally adjusted for parity. Adjusting for parity in other models made no appreciable difference to estimates.

[b]Study specific tertiles (low, medium, high) were used for all models except for folic acid from supplementation, which used cut-points based on the folate recommended daily intake (RDI) (0mcg, <400mcg, 400+mcg). Dietary folate equivalents (DFEs) were used for measures which included a component of folic acid intake.
