# Supplementary material for: Folate Intake and Ovarian Cancer Risk among Women with Endometriosis: A Case–Control Study from the Ovarian Cancer Association Consortium
Source: Cancer Epidemiol Biomarkers Prev. 2023 May 23;32(8):1087–96. doi: 10.1158/1055-9965.EPI-23-0121 (PMC10390886; doi:10.1158/1055-9965.EPI-23-0121)
Supplement: Supplementary Table 4 — shows the association between glycemic index, glycemic load, grain intake and risk of ovarian cancer for women with and without endometriosis. [file epi-23-0121_supplementary_table_4_suppst4.pdf]

Supplementary Table 4: Association between glycemic index, glycemic load, grain intake and risk of ovarian cancer, women with and without endometriosis

	Endometriosis		Without endometriosis	
**Variable**	**Cases/controls**	**OR (95% CI)[a]**	**Cases/controls**	**OR (95% CI)[a]**
**Glycemic index**				
Low	65/56	1.00 (ref)	543/873	1.00 (ref)
Medium	77/59	1.12 (0.66-1.90)	601/803	1.23 (1.05-1.43)
High	55/57	0.97 (0.55-1.70)	629/797	1.31 (1.13-1.53)
**Glycemic load**				
Low	76/66	1.00 (ref)	681/1043	1.00 (ref)
Medium	75/75	0.75 (0.46-1.23)	746/974	1.19 (1.03-1.36)
High	100/77	1.04 (0.64-1.70)	789/902	1.38 (1.20-1.59)
**Total grain intake**				
Low	176/181	1.00 (ref)	1737/2507	1.00 (ref)
Medium	196/196	0.81 (0.59-1.13)	1712/2527	0.95 (0.86-1.04)
High	198/181	0.81 (0.56-1.16)	1719/2510	0.95 (0.86-1.06)
**Refined grain intake**				
Low	174/200	1.00 (ref)	1756/2456	1.00 (ref)
Medium	219/174	1.26 (0.93-1.71)	1736/2532	0.92 (0.84-1.00)
High	177/184	0.89 (0.64-1.22)	1675/2555	0.85 (0.78-0.94)
**Whole grain intake**				
Low	176/186	1.00 (ref)	1704/2542	1.00 (ref)
Medium	193/188	0.90 (0.66-1.23)	1730/2517	1.04 (0.95-1.14)
High	201/184	0.91 (0.64-1.28)	1733/2485	1.07 (0.97-1.18)

Abbreviations: CI, confidence interval; OR, odds ratio.
[a]All models were adjusted for age (10 year age groups), log(energy intake), parity, dietary folate intake and stratified by site. Study specific tertiles (low, medium, high) were used for all models.
