# Supplementary material for: Folate Intake and Ovarian Cancer Risk among Women with Endometriosis: A Case–Control Study from the Ovarian Cancer Association Consortium
Source: Cancer Epidemiol Biomarkers Prev. 2023 May 23;32(8):1087–96. doi: 10.1158/1055-9965.EPI-23-0121 (PMC10390886; doi:10.1158/1055-9965.EPI-23-0121)
Supplement: Supplementary Table 5 — shows the results from the different Mendelian randomization methods investigating the association between genetically predicted folate and risk of ovarian cancer, by endometriosis status [file epi-23-0121_supplementary_table_5_suppst5.pdf]

Supplementary Table 5: The association between genetically-predicted folate and risk of ovarian cancer, women with and without endometriosis – comparison of different Mendelian randomization methods

	Odds ratio (95% CI)[a]	
**Method**	**Endometriosis**	**Without endometriosis**
IVW	2.22 (0.80-6.17)	0.90 (0.60-1.34)
Weighted median	1.83 (0.55-6.08)	0.95 (0.67-1.34)
Simple median	2.73 (0.76-9.85)	0.94 (0.67-1.32)
MR-Egger	0.07 (0.0-13.78)	1.19 (0.10-14.28)

Abbreviations: CI, confidence interval; IVW, inverse-variance weighted.
[a]Odds ratios were estimated using four SNPs that were associated with serum folate levels in the largest published GWAS to date.
